# Supplementary material for: Integrative Methylome and Transcriptome Characterization Identifies SERINC2 as a Tumor-Driven Gene for Papillary Thyroid Carcinoma
Source: Cancers (Basel). 2022 Dec 30;15(1):243. doi: 10.3390/cancers15010243 (PMC9818177; doi:10.3390/cancers15010243)
Supplement: Supplementary file 1 [file cancers-15-00243-s001.zip › Supplementary Materials.pdf]

Table of contents:

Supplementary Figures S1–S4

Supplementary Tables S1,S2,S4,S5（Supplementary Table S3 was uploaded alone)

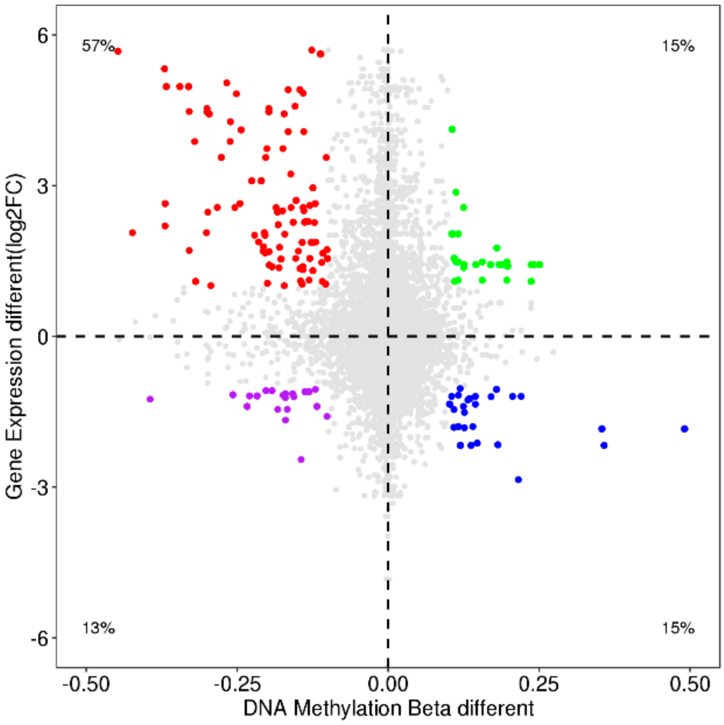

Figure S1. Volcano plot showing the integrative analysis of the methylation profile and transcriptome profile of PTC from TCGA.

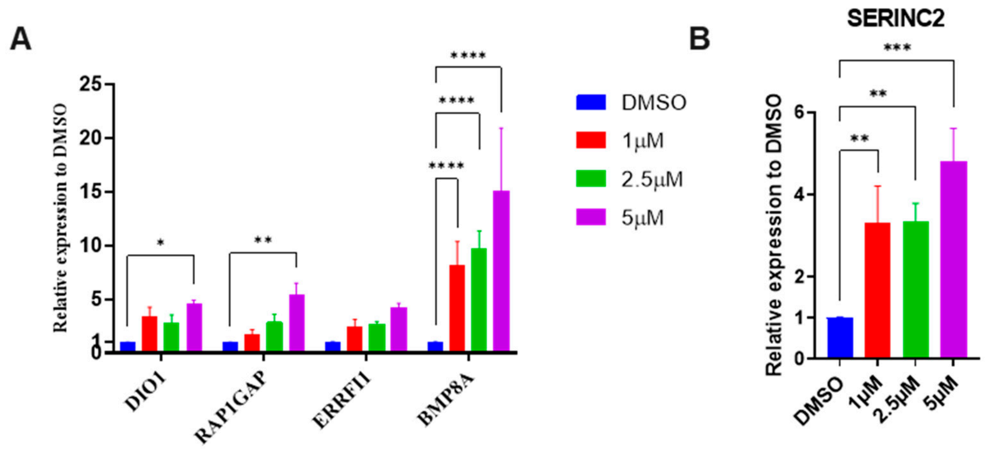

Figure S2. TPC-1 cells were treated with different doses (1, 2.5, and 5 μM) of 5-aza-2'deoxycytidine for 72 hours. **A.** Expression of the four chosen genes with high methylation detected by RT-qPCR was restored with increasing concentrations of 5-aza-2'deoxycytidine. **B.** Expression of SERINC2 detected by RT-qPCR. Quantitative results were analyzed by two-way ANOVA with $P \leq 0.05$ considered significant and are shown in the bar graph as mean ± SEM. *$P < 0.05$, **$P < 0.01$, ***$P < 0.005$ and ****$P < 0.001$.

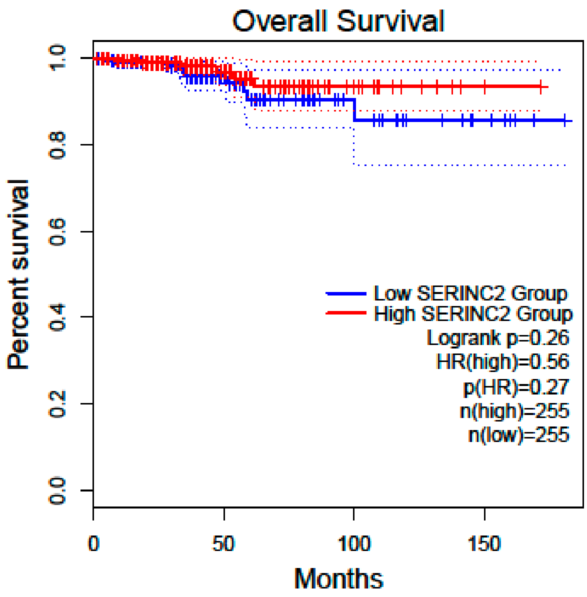

Figure S3. Overall survival analysis of thyroid carcinoma patients grouped by expression of SERINC2.

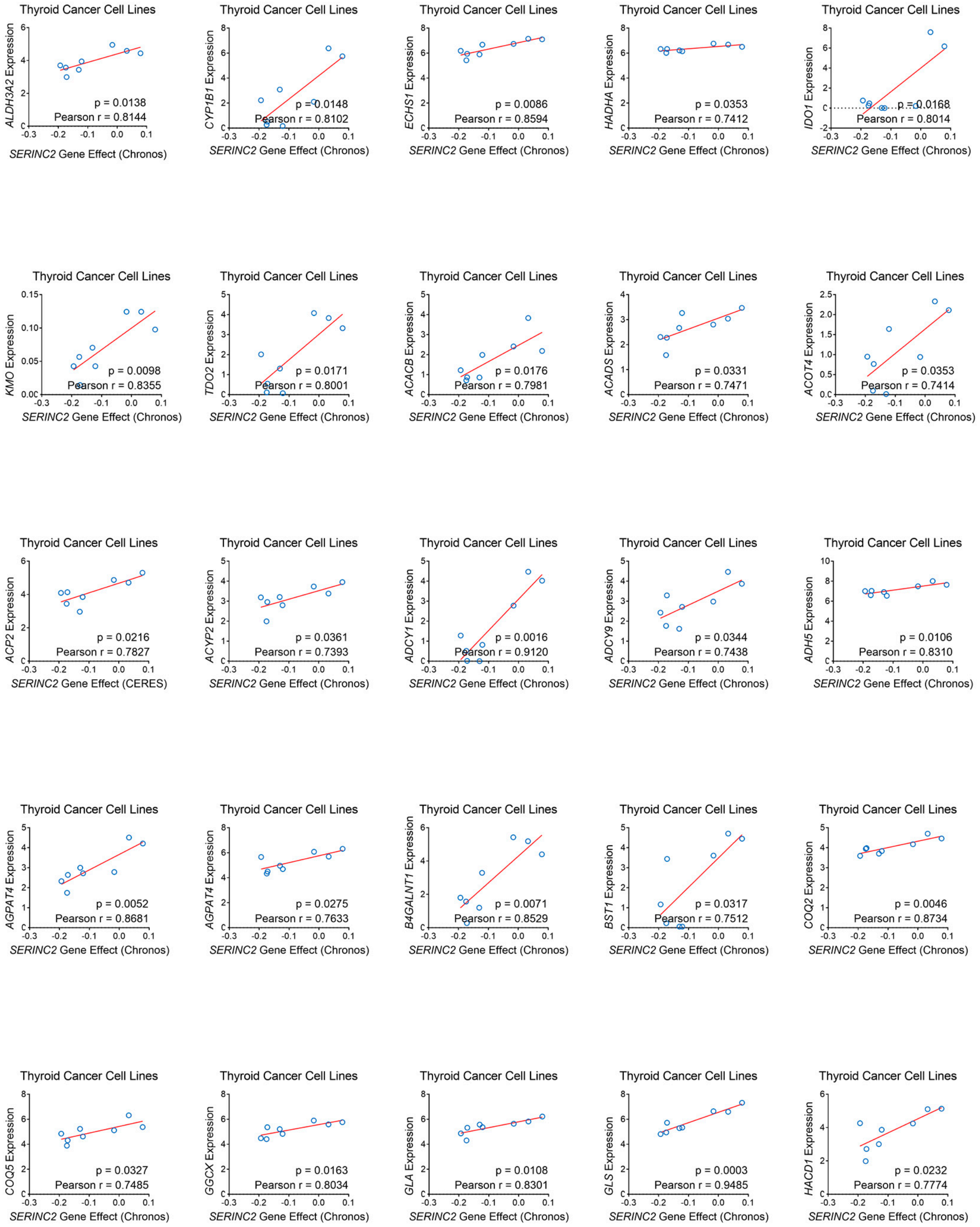

Thyroid Cancer Cell Lines
ALDH3A2 Expression
p = 0.0138
Pearson r = 0.8144
SERINC2 Gene Effect (Chronos)
Thyroid Cancer Cell Lines
CYP1B1 Expression
p = 0.0148
Pearson r = 0.8102
SERINC2 Gene Effect (Chronos)
Thyroid Cancer Cell Lines
ECHS1 Expression
p = 0.0086
Pearson r = 0.8594
SERINC2 Gene Effect (Chronos)
Thyroid Cancer Cell Lines
HADHA Expression
p = 0.0353
Pearson r = 0.7412
SERINC2 Gene Effect (Chronos)
Thyroid Cancer Cell Lines
IDO1 Expression
p = 0.0168
Pearson r = 0.8014
SERINC2 Gene Effect (Chronos)
Thyroid Cancer Cell Lines
KMO Expression
p = 0.0098
Pearson r = 0.8355
SERINC2 Gene Effect (Chronos)
Thyroid Cancer Cell Lines
TDO2 Expression
p = 0.0171
Pearson r = 0.8001
SERINC2 Gene Effect (Chronos)
Thyroid Cancer Cell Lines
ACACB Expression
p = 0.0176
Pearson r = 0.7981
SERINC2 Gene Effect (Chronos)
Thyroid Cancer Cell Lines
ACADS Expression
p = 0.0331
Pearson r = 0.7471
SERINC2 Gene Effect (Chronos)
Thyroid Cancer Cell Lines
ACOT4 Expression
p = 0.0353
Pearson r = 0.7414
SERINC2 Gene Effect (Chronos)
Thyroid Cancer Cell Lines
ACP2 Expression
p = 0.0216
Pearson r = 0.7827
SERINC2 Gene Effect (CERES)
Thyroid Cancer Cell Lines
ACYP2 Expression
p = 0.0361
Pearson r = 0.7393
SERINC2 Gene Effect (Chronos)
Thyroid Cancer Cell Lines
ADCY1 Expression
p = 0.0016
Pearson r = 0.9120
SERINC2 Gene Effect (Chronos)
Thyroid Cancer Cell Lines
ADCY9 Expression
p = 0.0344
Pearson r = 0.7438
SERINC2 Gene Effect (Chronos)
Thyroid Cancer Cell Lines
ADH5 Expression
p = 0.0106
Pearson r = 0.8310
SERINC2 Gene Effect (Chronos)
Thyroid Cancer Cell Lines
AGPAT4 Expression
p = 0.0052
Pearson r = 0.8681
SERINC2 Gene Effect (Chronos)
Thyroid Cancer Cell Lines
AGPAT4 Expression
p = 0.0275
Pearson r = 0.7633
SERINC2 Gene Effect (Chronos)
Thyroid Cancer Cell Lines
B4GALNT1 Expression
p = 0.0071
Pearson r = 0.8529
SERINC2 Gene Effect (Chronos)
Thyroid Cancer Cell Lines
BST1 Expression
p = 0.0317
Pearson r = 0.7512
SERINC2 Gene Effect (Chronos)
Thyroid Cancer Cell Lines
COQ2 Expression
p = 0.0046
Pearson r = 0.8734
SERINC2 Gene Effect (Chronos)
Thyroid Cancer Cell Lines
COQ5 Expression
p = 0.0327
Pearson r = 0.7485
SERINC2 Gene Effect (Chronos)
Thyroid Cancer Cell Lines
GGCX Expression
p = 0.0163
Pearson r = 0.8034
SERINC2 Gene Effect (Chronos)
Thyroid Cancer Cell Lines
GLA Expression
p = 0.0108
Pearson r = 0.8301
SERINC2 Gene Effect (Chronos)
Thyroid Cancer Cell Lines
GLS Expression
p = 0.0003
Pearson r = 0.9485
SERINC2 Gene Effect (Chronos)
Thyroid Cancer Cell Lines
HACD1 Expression
p = 0.0232
Pearson r = 0.7774
SERINC2 Gene Effect (Chronos)

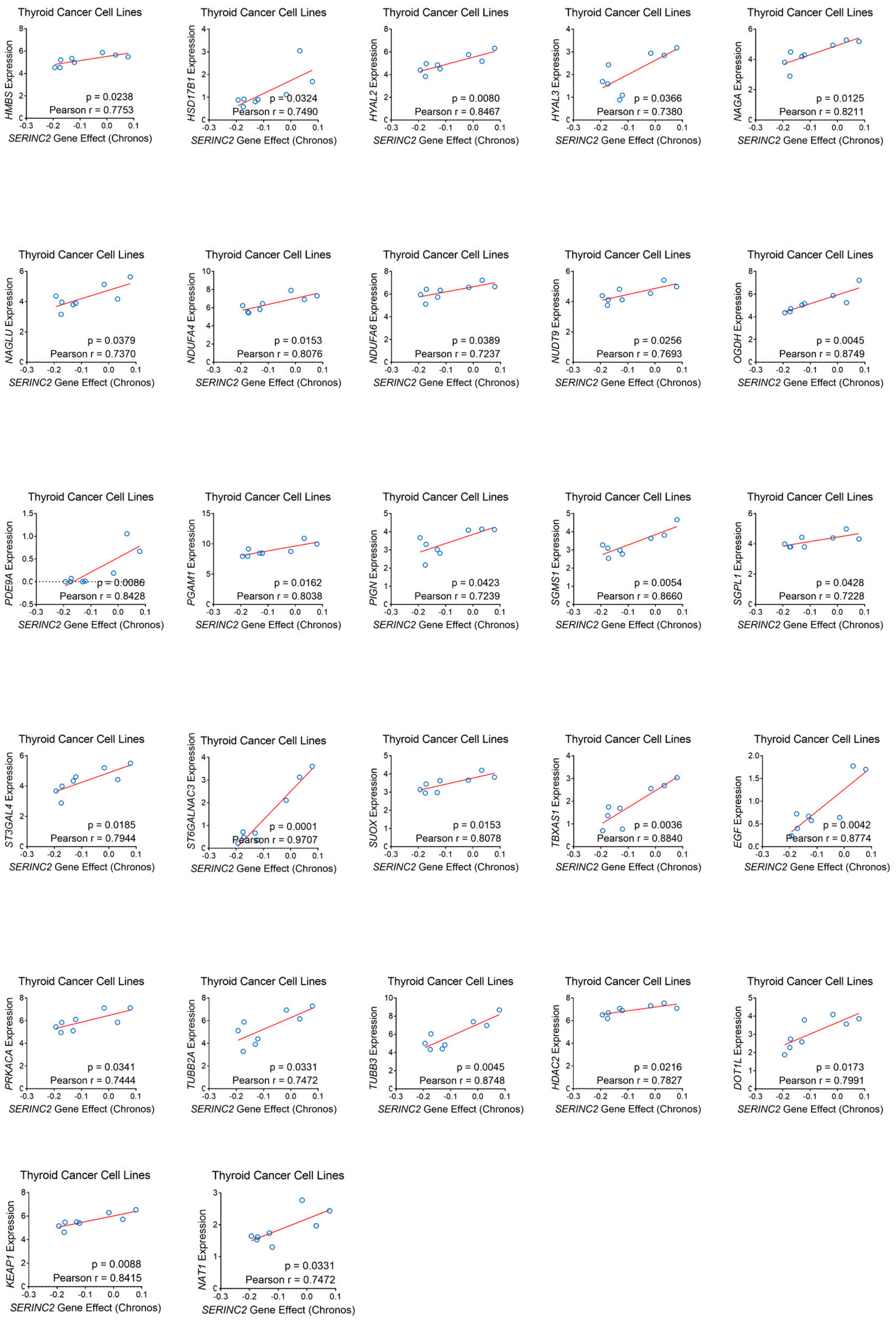
Thyroid Cancer Cell Lines
HMBS Expression
p = 0.0238
Pearson r = 0.7753
SERINC2 Gene Effect (Chronos)
Thyroid Cancer Cell Lines
HSD17B1 Expression
p = 0.0324
Pearson r = 0.7490
SERINC2 Gene Effect (Chronos)
Thyroid Cancer Cell Lines
HYAL2 Expression
p = 0.0080
Pearson r = 0.8467
SERINC2 Gene Effect (Chronos)
Thyroid Cancer Cell Lines
HYAL3 Expression
p = 0.0366
Pearson r = 0.7380
SERINC2 Gene Effect (Chronos)
Thyroid Cancer Cell Lines
NAGA Expression
p = 0.0125
Pearson r = 0.8211
SERINC2 Gene Effect (Chronos)
Thyroid Cancer Cell Lines
NAGLU Expression
p = 0.0379
Pearson r = 0.7370
SERINC2 Gene Effect (Chronos)
Thyroid Cancer Cell Lines
NDUFA4 Expression
p = 0.0153
Pearson r = 0.8076
SERINC2 Gene Effect (Chronos)
Thyroid Cancer Cell Lines
NDUFA6 Expression
p = 0.0389
Pearson r = 0.7237
SERINC2 Gene Effect (Chronos)
Thyroid Cancer Cell Lines
NUDT9 Expression
p = 0.0256
Pearson r = 0.7693
SERINC2 Gene Effect (Chronos)
Thyroid Cancer Cell Lines
OGDH Expression
p = 0.0045
Pearson r = 0.8749
SERINC2 Gene Effect (Chronos)
Thyroid Cancer Cell Lines
PDE9A Expression
p = 0.0086
Pearson r = 0.8428
SERINC2 Gene Effect (Chronos)
Thyroid Cancer Cell Lines
PGAM1 Expression
p = 0.0162
Pearson r = 0.8038
SERINC2 Gene Effect (Chronos)
Thyroid Cancer Cell Lines
PIGN Expression
p = 0.0423
Pearson r = 0.7239
SERINC2 Gene Effect (Chronos)
Thyroid Cancer Cell Lines
SGMS1 Expression
p = 0.0054
Pearson r = 0.8660
SERINC2 Gene Effect (Chronos)
Thyroid Cancer Cell Lines
SGPL1 Expression
p = 0.0428
Pearson r = 0.7228
SERINC2 Gene Effect (Chronos)
Thyroid Cancer Cell Lines
ST3GAL4 Expression
p = 0.0185
Pearson r = 0.7944
SERINC2 Gene Effect (Chronos)
Thyroid Cancer Cell Lines
ST6GALNAC3 Expression
p = 0.0001
Pearson r = 0.9707
SERINC2 Gene Effect (Chronos)
Thyroid Cancer Cell Lines
SUOX Expression
p = 0.0153
Pearson r = 0.8078
SERINC2 Gene Effect (Chronos)
Thyroid Cancer Cell Lines
TBXAS1 Expression
p = 0.0036
Pearson r = 0.8840
SERINC2 Gene Effect (Chronos)
Thyroid Cancer Cell Lines
EGF Expression
p = 0.0042
Pearson r = 0.8774
SERINC2 Gene Effect (Chronos)
Thyroid Cancer Cell Lines
PRKACA Expression
p = 0.0341
Pearson r = 0.7444
SERINC2 Gene Effect (Chronos)
Thyroid Cancer Cell Lines
TUBB2A Expression
p = 0.0331
Pearson r = 0.7472
SERINC2 Gene Effect (Chronos)
Thyroid Cancer Cell Lines
TUBB3 Expression
p = 0.0045
Pearson r = 0.8748
SERINC2 Gene Effect (Chronos)
Thyroid Cancer Cell Lines
HDAC2 Expression
p = 0.0216
Pearson r = 0.7827
SERINC2 Gene Effect (Chronos)
Thyroid Cancer Cell Lines
DOT1L Expression
p = 0.0173
Pearson r = 0.7991
SERINC2 Gene Effect (Chronos)
Thyroid Cancer Cell Lines
KEAP1 Expression
p = 0.0088
Pearson r = 0.8415
SERINC2 Gene Effect (Chronos)
Thyroid Cancer Cell Lines
NAT1 Expression
p = 0.0331
Pearson r = 0.7472
SERINC2 Gene Effect (Chronos)

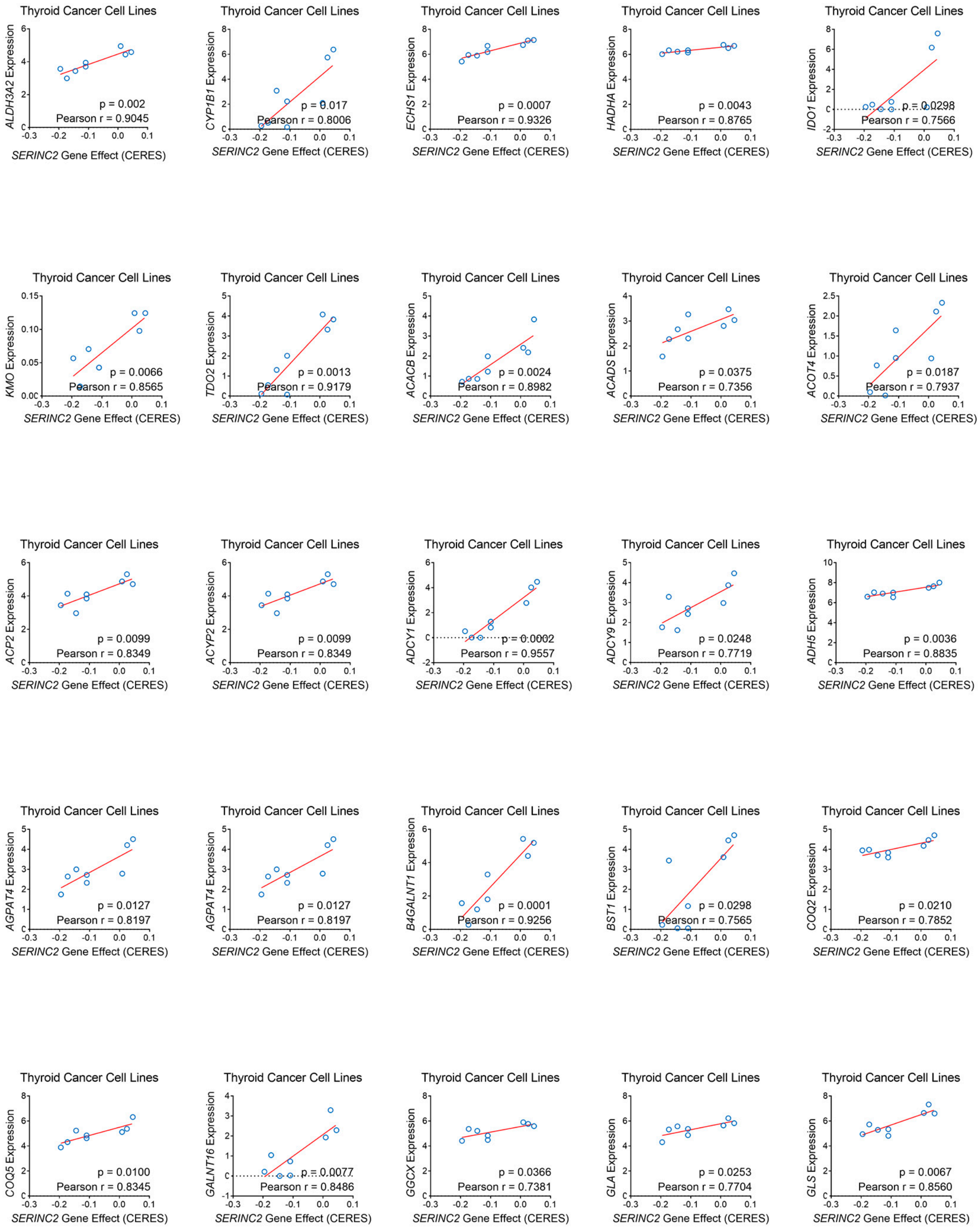
Thyroid Cancer Cell Lines
ALDH3A2 Expression
p = 0.002
Pearson r = 0.9045
SERINC2 Gene Effect (CERES)
Thyroid Cancer Cell Lines
CYP1B1 Expression
p = 0.017
Pearson r = 0.8006
SERINC2 Gene Effect (CERES)
Thyroid Cancer Cell Lines
ECHS1 Expression
p = 0.0007
Pearson r = 0.9326
SERINC2 Gene Effect (CERES)
Thyroid Cancer Cell Lines
HADHA Expression
p = 0.0043
Pearson r = 0.8765
SERINC2 Gene Effect (CERES)
Thyroid Cancer Cell Lines
IDO1 Expression
p = 0.0298
Pearson r = 0.7566
SERINC2 Gene Effect (CERES)
Thyroid Cancer Cell Lines
KMO Expression
p = 0.0066
Pearson r = 0.8565
SERINC2 Gene Effect (CERES)
Thyroid Cancer Cell Lines
TDO2 Expression
p = 0.0013
Pearson r = 0.9179
SERINC2 Gene Effect (CERES)
Thyroid Cancer Cell Lines
ACACB Expression
p = 0.0024
Pearson r = 0.8982
SERINC2 Gene Effect (CERES)
Thyroid Cancer Cell Lines
ACADS Expression
p = 0.0375
Pearson r = 0.7356
SERINC2 Gene Effect (CERES)
Thyroid Cancer Cell Lines
ACOT4 Expression
p = 0.0187
Pearson r = 0.7937
SERINC2 Gene Effect (CERES)
Thyroid Cancer Cell Lines
ACP2 Expression
p = 0.0099
Pearson r = 0.8349
SERINC2 Gene Effect (CERES)
Thyroid Cancer Cell Lines
ACYP2 Expression
p = 0.0099
Pearson r = 0.8349
SERINC2 Gene Effect (CERES)
Thyroid Cancer Cell Lines
ADCY1 Expression
p = 0.0002
Pearson r = 0.9557
SERINC2 Gene Effect (CERES)
Thyroid Cancer Cell Lines
ADCY9 Expression
p = 0.0248
Pearson r = 0.7719
SERINC2 Gene Effect (CERES)
Thyroid Cancer Cell Lines
ADH5 Expression
p = 0.0036
Pearson r = 0.8835
SERINC2 Gene Effect (CERES)
Thyroid Cancer Cell Lines
AGPAT4 Expression
p = 0.0127
Pearson r = 0.8197
SERINC2 Gene Effect (CERES)
Thyroid Cancer Cell Lines
AGPAT4 Expression
p = 0.0127
Pearson r = 0.8197
SERINC2 Gene Effect (CERES)
Thyroid Cancer Cell Lines
B4GALNT1 Expression
p = 0.0001
Pearson r = 0.9256
SERINC2 Gene Effect (CERES)
Thyroid Cancer Cell Lines
BST1 Expression
p = 0.0298
Pearson r = 0.7565
SERINC2 Gene Effect (CERES)
Thyroid Cancer Cell Lines
COQ2 Expression
p = 0.0210
Pearson r = 0.7852
SERINC2 Gene Effect (CERES)
Thyroid Cancer Cell Lines
COQ5 Expression
p = 0.0100
Pearson r = 0.8345
SERINC2 Gene Effect (CERES)
Thyroid Cancer Cell Lines
GALNT16 Expression
p = 0.0077
Pearson r = 0.8486
SERINC2 Gene Effect (CERES)
Thyroid Cancer Cell Lines
GGCX Expression
p = 0.0366
Pearson r = 0.7381
SERINC2 Gene Effect (CERES)
Thyroid Cancer Cell Lines
GLA Expression
p = 0.0253
Pearson r = 0.7704
SERINC2 Gene Effect (CERES)
Thyroid Cancer Cell Lines
GLS Expression
p = 0.0067
Pearson r = 0.8560
SERINC2 Gene Effect (CERES)

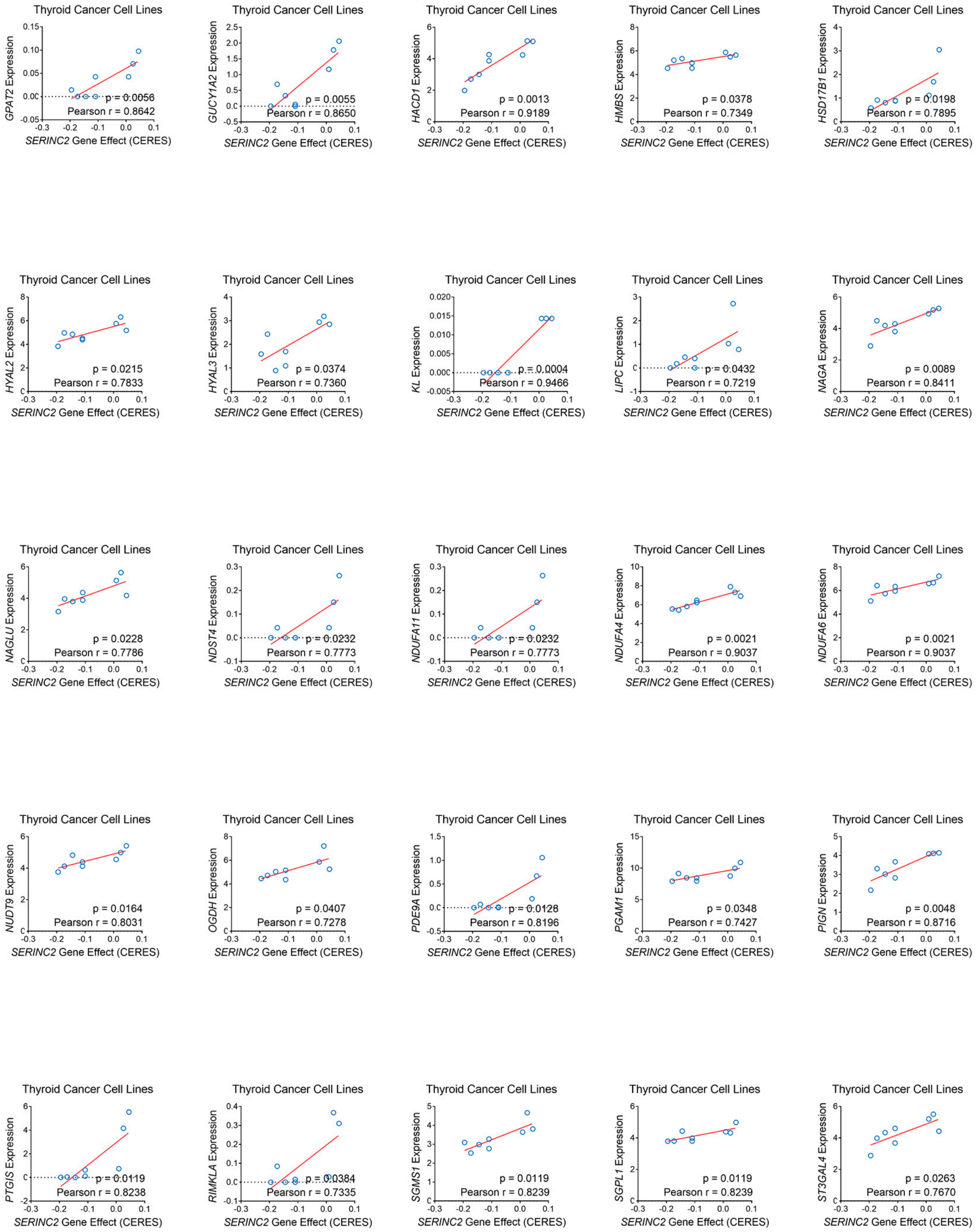

Thyroid Cancer Cell Lines
GPAT2 Expression
p = 0.0056
Pearson r = 0.8642
SERINC2 Gene Effect (CERES)
Thyroid Cancer Cell Lines
GUCY1A2 Expression
p = 0.0055
Pearson r = 0.8650
SERINC2 Gene Effect (CERES)
Thyroid Cancer Cell Lines
HACD1 Expression
p = 0.0013
Pearson r = 0.9189
SERINC2 Gene Effect (CERES)
Thyroid Cancer Cell Lines
HMBS Expression
p = 0.0378
Pearson r = 0.7349
SERINC2 Gene Effect (CERES)
Thyroid Cancer Cell Lines
HSD17B1 Expression
p = 0.0198
Pearson r = 0.7895
SERINC2 Gene Effect (CERES)
Thyroid Cancer Cell Lines
HYAL2 Expression
p = 0.0215
Pearson r = 0.7833
SERINC2 Gene Effect (CERES)
Thyroid Cancer Cell Lines
HYAL3 Expression
p = 0.0374
Pearson r = 0.7360
SERINC2 Gene Effect (CERES)
Thyroid Cancer Cell Lines
KL Expression
p = 0.0004
Pearson r = 0.9466
SERINC2 Gene Effect (CERES)
Thyroid Cancer Cell Lines
LIPC Expression
p = 0.0432
Pearson r = 0.7219
SERINC2 Gene Effect (CERES)
Thyroid Cancer Cell Lines
NAGA Expression
p = 0.0089
Pearson r = 0.8411
SERINC2 Gene Effect (CERES)
Thyroid Cancer Cell Lines
NAGLU Expression
p = 0.0228
Pearson r = 0.7786
SERINC2 Gene Effect (CERES)
Thyroid Cancer Cell Lines
NDST4 Expression
p = 0.0232
Pearson r = 0.7773
SERINC2 Gene Effect (CERES)
Thyroid Cancer Cell Lines
NDUFA11 Expression
p = 0.0232
Pearson r = 0.7773
SERINC2 Gene Effect (CERES)
Thyroid Cancer Cell Lines
NDUFA4 Expression
p = 0.0021
Pearson r = 0.9037
SERINC2 Gene Effect (CERES)
Thyroid Cancer Cell Lines
NDUFA6 Expression
p = 0.0021
Pearson r = 0.9037
SERINC2 Gene Effect (CERES)
Thyroid Cancer Cell Lines
NUDT9 Expression
p = 0.0164
Pearson r = 0.8031
SERINC2 Gene Effect (CERES)
Thyroid Cancer Cell Lines
OGDH Expression
p = 0.0407
Pearson r = 0.7278
SERINC2 Gene Effect (CERES)
Thyroid Cancer Cell Lines
PDE9A Expression
p = 0.0128
Pearson r = 0.8196
SERINC2 Gene Effect (CERES)
Thyroid Cancer Cell Lines
PGAM1 Expression
p = 0.0348
Pearson r = 0.7427
SERINC2 Gene Effect (CERES)
Thyroid Cancer Cell Lines
PIGN Expression
p = 0.0048
Pearson r = 0.8716
SERINC2 Gene Effect (CERES)
Thyroid Cancer Cell Lines
PTGIS Expression
p = 0.0119
Pearson r = 0.8238
SERINC2 Gene Effect (CERES)
Thyroid Cancer Cell Lines
RIMKLA Expression
p = 0.0384
Pearson r = 0.7335
SERINC2 Gene Effect (CERES)
Thyroid Cancer Cell Lines
SGMS1 Expression
p = 0.0119
Pearson r = 0.8239
SERINC2 Gene Effect (CERES)
Thyroid Cancer Cell Lines
SGPL1 Expression
p = 0.0119
Pearson r = 0.8239
SERINC2 Gene Effect (CERES)
Thyroid Cancer Cell Lines
ST3GAL4 Expression
p = 0.0263
Pearson r = 0.7670
SERINC2 Gene Effect (CERES)

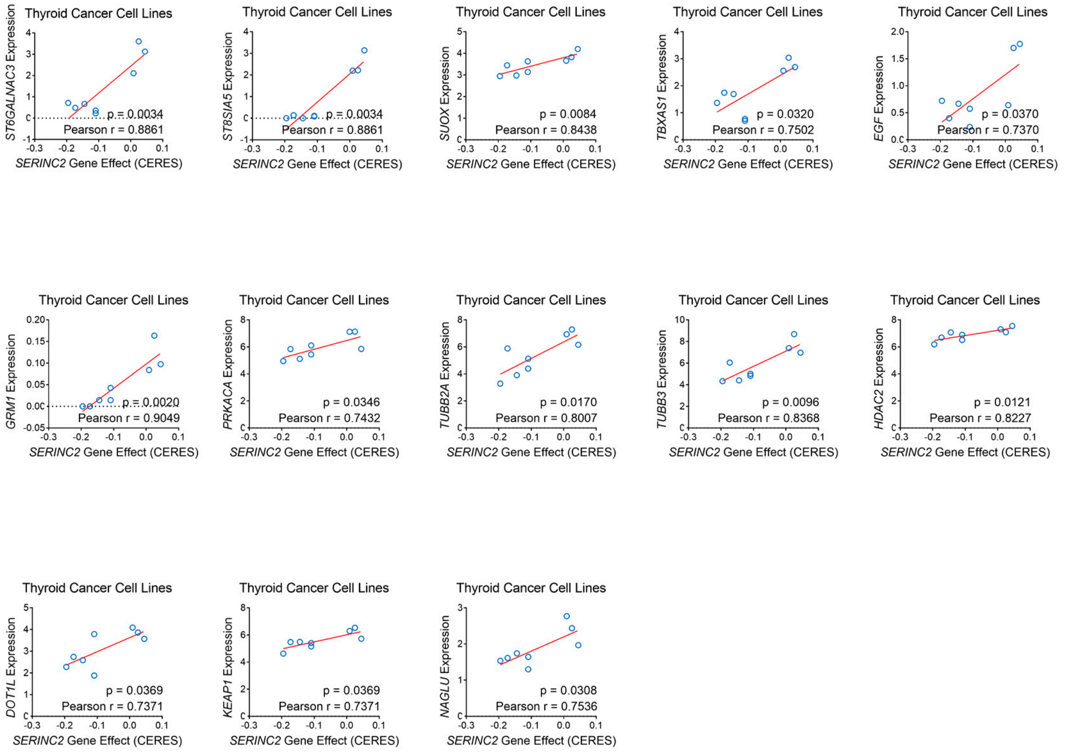

Figure S4. Dependency of selected co-expression genes of SERINC2 from Depmap.

Table S1. The characteristics of the patients whose samples were used in this study.

Group	Age	Sex	Tumor_size (cm)	ETE*	T stage	LN_metastasis	distant_metastasis
BTL	35	male	4	0	T0	0	0
BTL	43	female	4	0	T0	0	0
BTL	53	male	4.5	0	T0	0	0
BTL	54	female	4.5	0	T0	0	0
BTL	70	male	4.5	0	T0	0	0
BTL	61	female	5	0	T0	0	0
PTC	25	female	2.5	1	T2	1	0
PTC	58	male	1.5	0	T1b	0	0
PTC	29	male	1.7	0	T1b	0	0
PTC	70	male	2.7	0	T2	0	0
PTC	26	male	1.5	0	T1b	0	0

*TET, extrathyroidal extension

Table S2. The sequences of primers used in this study.

SERINC2-qPCR-F	GCCTTTGACAACGAGCAGGA
SERINC2-qPCR-R	CCAGTTGGTGAGCGTCATCA
shSERINC2#1-F	CCGGGTGCTGGTGTCCATCATTATGCTCGAGCATAATGATGGACACC AGCACTTTTTG
shSERINC2#1-R	AATTCAAAAAGTGCTGGTGTCCATCATTATGCTCGAGCATAATGATGG ACACCAGCAC
shSERINC2#2-F	CCGGTGGTCAGCCCTATCCAGTATCCTCGAGGATACTGGATAGGGCT GACCATTTTTG
shSERINC2#2-R	AATTCAAAAATGGTCAGCCCTATCCAGTATCCTCGAGGATACTGGATA GGGCTGACCA

Table S4. Metabolic pathway-related genes with $\log_2|FC|>1$ in PTC samples.

Gene name
CYP1B1, GSTM2, GSTM4, HGD, LIPH, LRP2, MAT2A, PCYT1A, PLA2R1, PRKACB, RPL14, SDC1, SERINC2, TPO

Table S5. Genes selected with $\log_2|FC|>1$ and $|\Delta\beta|>0.1$ in PTC samples and $\log_2|FC|>1$ in TCGA dataset.

	Gene name
hypermethylated and down-regulated	KCNK2, TPO, BMP8A, EFEMP1, PLSCR4, ESRRG, RAP1GAP, DIO1, PAX8, ID3
hypomethylated and up-regulated	XDH, TM4SF1, TACSTD2, S100A4, CHI3L1, SFTPB, CLDN16, LIPH, DPP4, S100A9, ADORA1, SERINC2, XPR1, FABP3, S100A11, IGF2BP2, S100A10, CCL20, CYP1B1, CLDN1, CHIT1, IL1RAP, LEMD1, NRP2, CPA3, QPCT, PELI1
